# Ætiology of Dental Caries

**Published:** 1874-10

**Authors:** A. W. Harlan

**Affiliations:** Chicago.


AMERICAN JOURNAL

OF

### DENTAL SCIENCE.

Vol. VIII. THIRD SERIES--OCTOBER, 1874. No. 6.

### ARTICLE I.

### *Ætiology of Dental Caries.*

BY A. W. HARLAN, CHICAGO.

Read before the Illinois State Dental Society.

From the earliest period in the history of dental science there has been diverse opinions as to the cause of caries of the teeth, and up to the present time the vexed question has not been settled. One asserts that decay is caused by the action of acids entirely; another maintains that acids are only the commencement of it, and afterwards a parasitic plant is wholly the cause of the subsequent changes observed; still another observes the loss of the lime salts beneath a perfect gold filling. The same writer (Dr. H. S. Chase,) instances the absorption of dentine beneath what he terms necrosed enamel from pressure caused by a contiguous tooth; the pressure causing the circulation of the blood plasma to cease in the basal substance of the enamel; the discoloration taking place as a natural consequence; and the irritation simultaneous with the necrotic process causing the vital movement in the dentine which carried away its lime salts.

Thus we have three different theories upon the immediate or exciting causes of decay of the teeth ; the chemical, the parasitic, and the vital. Before expressing an opinion as to what agents cause the destruction of the teeth we will see what the predisposing causes are :

In examining authorities on the subject we find that " the power of teeth to resist decay is sometimes weakened by a change brought about in their physical condition through the agency of certain remote causes, such as the profuse administration of mercury, the existence of exanthematous diseases in infancy and early life, and all severe constitutional disorders." Harris and Garretson believe that there is an hereditary tendency in certain teeth to decay, even to the transmission of the shapes of the defective teeth of the parents. This might be true, if the same condition otherwise obtained in the mouths of both parent and child, but on the reversal of that condition it is obvious that decay would not be the result of hereditary transmission. Lack of exercise is supposed, by some, to be a predisposing cause of decay ; imperfect structural formation, pits, fissures, depressions and cracks in the enamel, affording a place of lodgment for food, acids, etc., are all predisposing causes. The six-year molars are often what Tomes describes as in a honey-combed condition, and are therefore peculiarly liable to decay. The changes in temperature from hot to cold do not, according to Dr. Flagg, check the enamel, thus facilitating decay, but diminished vitality is conceded to be the result of long continued alterations in temperature from hot to cold.

Mr. Oakely Coales in a little work called " Notes on Dental Pathology," says : " The experience of many accurately collected and carefully compared, will, I believe, show that it is impossible to assign any single process or action as the cause of decay in the dental organs. When we remember that the mouth is the chamber through which passes all the nutrition of the body, solid fluid and gaseous, and that it is liable to be acted upon by all the various conditions and temperatures in which the several forms of nutrition may be taken in, there seems fair ground for coming to the conclusion that all these, severaily or in combination, may produce that condition, which we know in the teeth as caries. I would not shut out, as predisposing states, mechanical pressure and irritation going on to such an extent as to cause disintegration of the tooth substance ; but I would not have them confounded with the immediate cause of decay."

"Looking at the facts that are constantly being brought under the practitioner's notice of cases where the patient tells us that up to the time of some illness or other there was no decay of the teeth, *it seems irresistible that we must look for the cause of caries within, and not without the tooth.*"

Further on he says: "Is it not reasonable to suppose that in all cases of caries an organic change of the tooth itself takes place, forming the first stage of decay? May we not go further and say that in many instances we can assign the cause as a want of balance between the organic and inorganic constituents of the tooth, and that in others it is the general impaired nutrition of a local, or, it may be, a result of character?"

"If it can be clearly shown that the actual and immediate cause of caries in the teeth is from within, then we have more certain data for going upon in the treatment of this form of disease."

I suppose that inflammation of the gums consequent upon a scrofulous condition of the general system is a predisposing cause of caries. Many other causes that are well known, such as stomatitis, dyspepsia, etc., might be enumerated, but the above are sufficient for our purpose. This brings us to the immediate or exciting cause of caries. The experiments of Westport, Allport, Mantegazza, Magitot, and others, agree in showing that vegetable and mineral acids, however, it is said, do not all destroy the same tissues—that is, some act upon the enamel, others upon the dentine and cementum. Leber and Rottenstein state that all acids are capable of destroying any part of a tooth if you will give them time enough. In the experiments out of the mouth they think also, that decalcification of the dentine takes place simultaneously with that of the enamel, only that it is not so readily observed. I see no reason why this may not be so. As Todd and Bowman observe, that canals exist between the enamel prisma, *why may not the acid penetrate these minute canals, and the work of destruction progress within at the same time that softening of the enamel is taking place from without?* Tomes found these canals in the teeth of young animals. Czermack believes that he has seen very numerous delicate enamel tubules arrayed in close series. We all know how soon our teeth can be "set on edge" by eating a lemon. May not this account also for a phenomenon observed by Prof. Chase in his paper of last year? We find two teeth very close together, something lodges between them long enough to decompose and give off an acid which enters the canals referred to; the work of destruction has commenced within, and by the time we suspect caries, from the black or brownish appearance of the enamel, *there has been a softening of dentine beneath this dark spot of enamel*, but vital action did not cause it, *it was the penetration of the acid through the canals between the enamel prisma.* After cutting through the black or brownish spots with the "shining surfaces" do we find a portion of the calcareous salts to have disappeared? I answer, not at all, but we do find a pulverulent mass that we can blow out of the cavity by an air syringe, which in my opinion, from actual observation, is the disintegrated lime salts.

These cases are very rare; I had seen but one such before having listened to the reading of Dr. Chase's paper, and could not account for it. After my return from Rock Island last year, I requested the privilege of cutting into another tooth of like character (as above described) in the same patient's mouth; he consented. It was exactly similar in appearance to the one first operated upon, and is described in the paper to which reference has been made. Since that time, I have observed four other cases of the same nature, save in the appearance of the color, which has varied between the black and brown. The vital theory has been pretty severely handled by Mr. Chas. Tomes in an appendix to the new edition "Tomes' Dental Surgery," to which I refer you; further on I shall have occasion to recur to it slightly.

The work of Leber & Rottenstein has been written to support the parasitic theory; the *Leptothrix Buccalis* is the parasite which, according to them, performs the work of destruction. After the enamel has become roughened by the action of an acid, the fungus finds lodgment and commences the envelopment of minute fragments of the decalcified enamel, "and contributes by their prodigious proliferation, to their destruction." Their opinion of the cause of caries of the enamel is, that "by the action of an acid the enamel becomes porous at some point, and loses its normal consistence. At the same time there is seen to appear a brown color, in consequence of the change which has taken place in its organic structure. There is formed at the surface a bed of leptothrix, which probably penetrates the dental cuticle—if it still exists—and destroys it; chinks and fissures are opened in the enamel, which has become less consistent, acid liquids and granulations of leptothrix penetrate there, while minute fragments of enamel become detached, and are promptly enveloped by the elements of the leptothrix, which, joined to the continued action of the acid, hastens the dissolution."

They attribute the brownish color of the enamel to the decomposition of the organic portions of the tooth; according to their observations the dentine is destroyed in what they term two periods. First—The preparatory work of caries. Second—The period of destruction. Of the first they observe that "the softened dentine of a brownish color and of a feeble consistence, situated under a carious surface, presents, after removal of the masses of leptothrix which penetrate there, peculiar changes in the dental canaliculi. The changes become, in general, more and more perceptible as we proceed from the depths of the dentine to its surface. It is easy to see in cross sections of teeth *that the canaliculi become very gradually longer, till they reach a considerable size, by the accumulation in their interior of a finely granular substance.*" This substance is granular leptothrix. The walls of the canaliculi continue to dilate until "it may happen that they cease to be visible." M. Neuman, as an evidence of the power of the dentine to resist that condition known as caries, says that the thickening of the walls of the tubes is a real thickening of the canaliculi at the expense of the matrix, and that the fibres participate in the process, the canals ultimately becoming obliterated ; he believes that in one instance he saw calcification of the fibrils.

Tomes, on the same subject, says that "Some such explanation as the following may very possibly be the true one : A solvent fluid gaining access to the tubes, effects the decalcification, complete or partial, of the matrix immediately round the tube ; as the refractive index of the dentinal sheaths differs little, if at all, from that of the surrounding decalcified matrix, they are not visible on transverse section ; but, on account of the great power of resistance, they ultimately become isolated by the wasting away of the matrix around them." You see from the above the different appearances observed in the preparatory work of caries.

We pass to the second, the "period of destruction." Leber and Rottenstein say : "If we examine the disorganized substance which covers the superfices of a carious cavity, and which, moreover, presents an acid reaction, we are truly astonished at the quantity of leptothrix which are found there. The superficial layers, with the exception of some particles of food, are formed exclusively by the granular masses, and the filaments of leptothrix." They observe farther "that minute fragments of carious dentine, of a brown color, are enveloped and united by masses of leptothrix, above all, granular leptothrix. In the layers which lie still deeper, as the volume of the dentine increases, the leptothrix diminishes in such a proportion that the dentine forms the principal part. We see, then, in the carious portions, colored brown, irregular chinks and interstices filled with leptothrix and its elements. Further: "According to our observations we cannot refuse to admit that the proliferation of the fungus plays an important part in the decomposition of the dental tissues." To sum up they believe that the action of acids upon the teeth affords a place of lodgment for the leptothrix buccallis; that the envelopment of minute fragments of decalcified enamel by this fungus soon effects an opening to the dentine, when, the acid passing before, allows the elements of the fungus to assist in the destruction of the dentine, by entering the canaliculi, thus dilating them, and forcing the passage of the acid still deeper.

We all think that decalcification of the dentine takes place before the leptothrix enters the carious cavity. I think that this is true myself, and believe further that the leptothrix plays a small part in the production of caries, if any at all, for the following reason: Very often we find carious cavities situated on the buccal surfaces of molar teeth, and in connection with them an acid buccal mucus. Now, we are told by Gubler and others that the leptothrix is the frame work of tartar. We are informed also that the fungi seem to thrive in a neutral or slightly acid liquid; the filiments of the fungus lodge in these cavities and immediately there is commenced a deposition of tartar. From the very moment of the commencement of the deposition of tartar, decay ceases in these cavities; this condition is not a common one, but we have all noticed it. It has been said that these deposits of tartar in buccal cavities answered the purpose of a filling very well, if they were only smoothed off. I do not see how the parasite assists in the destruction of the teeth except in this manner, that is, by the envelopment of the disintegrated lime salts as rapidly as disintegration is effected by acids, thus helping to abstract them from the organic tissue much sooner than they would be washed out by the fluids of the mouth. That parasites assist in causing the decay of teeth, I am wholly prepared to deny, but in what manner specifically I await to be informed.

Regarding the theory that vital action assists in the production of dental caries, I believe that any thing or cause, which tends to keep the teeth from being nourished properly, would cease a weakness in the tooth structure, making it easier for the acids to act upon them. Without saying anything further of this I conclude that there is no proof of the existence of central caries.

There is no species of decay that I have met with where the organic tissue of the tooth seemed to be wanting, leaving a residue of lime salts and also a porous substance very chalky in color and consistence. Where enamel still stands around the cavity, it is of the same nature, very easy to pulverize after removing it; no strength in it at all as far as the decay has penetrated. This condition is oftenest met with in the mouths of persons with flaxen hair, light complexion, etc., and the teeth are usually of medium size, with a slight yellow tinge near the gums, gradually becoming lighter in color until the cutting edge is nearly reached, when there is seen an almost imperceptible shade of a bluish cast at the extreme points of the teeth; these teeth decay very early and with great rapidity; very often in the short space of three months large cavities are seen on the labial surfaces of the lower cuspids and bicuspids; test the mouth and we *do not* find an acid reaction, the fluids almost invariably are alkaline, occasionally we find the saliva neutral. We know that excessive alkalinity of the saliva *might* cause this condition, but *does* it? There is no stringy or ropy mucus in this class of cases. I leave the solution of this question to be brought out in the discussions. One thing more about the effects of alkalies upon the teeth. At the meeting of the "American Dental Association," at Put-in-Bay last year, Dr. T. O. Summers said with reference to the action of acids and alkalies upon the teeth in the mouth: "I am still of the opinion that the alkalies are the most destructive agents. They act upon the pabulum, taking away the base. The presence of acids does not prove that they are the cause of decay. There is nothing so destructive to animal life as the alkalies." Again in answer to a question he said: "The alkalies enter the blood vessels, coming directly in contact with the tooth substance through the local circulation, and so destroy the pabulum of which the tooth is formed." On being asked why it did not act upon the bones and other tissues, he said: "It goes into the blood at a point immediately surrounding the teeth through the corpuscles, and destroys the pabulum." This I leave also for your consideration.

In conclusion, I believe that we must look for the causes of caries almost wholly if not altogether in the vegetable and mineral acids; we find in nearly every mouth examined, an acid reaction, and where a structural defeat exists in the enamel, or any predisposing cause of decay is present in the mouth, there we will find that disintegration has commenced. Wherever teeth are very close together, so as to afford a place of lodgment for particles of food, etc., we find an evidence of the commencement of caries. Anything that tends to produce irritation of the gums, causing an acid condition, should receive appropriate treatment.

Dr. J. M. Whitney has recorded thefact of the introduction of acid fruits among the inhabitants of the Sandwich Islands; commenting upon it, he says: "So marked was the difference in the teeth of the skeletons that I could mark the time when fruits was introduced. In those fed on fruits there was one (tooth) in fifty decayed, in the older, (skeletons) one in a hundred."

It has been agreed by some that acids did not produce sufficient varieties of color in decay, to be accounted as the agents that work the destruction. I believe that my friend

Dr. Black, who is present, can account for the different colors observed in dental caries to the complete satisfaction of those who have doubted the sole effect of corrosive agents in the production of that condition known and described as caries of the teeth ; a condition at once the most direful and calamitous in its nature, and one that calls out our best efforts to arrest its ravages.

